# Efficacy and Safety of Remimazolam Versus Etomidate for Induction of General Anesthesia: Protocol for a Systematic Review and Meta-Analysis

**DOI:** 10.2196/55948

**Published:** 2024-06-12

**Authors:** Li Zhao, Yiping Guo, Xuelei Zhou, Wei Mao, Linlin Chen, Ying Xie, Linji Li

**Affiliations:** 1 Department of Anesthesiology The Second Clinical Medical College, North Sichuan Medical College Nanchong Central Hospital Nanchong China; 2 School of Humanities and Management Key Laboratory for Quality of Life and Psychological Assessment and Intervention Guangdong Medical University Dongguan China; 3 Nanchong Center for Disease Control and Prevention Nanchong China

**Keywords:** general anesthesia, anesthesia induction, postinduction hypotension, remimazolam, etomidate, meta-analysis

## Abstract

**Background:**

Postinduction hypotension (PIHO) is a hemodynamic abnormality commonly observed during the induction of general anesthesia. Etomidate is considered a safer drug for the induction of anesthesia because it has only minor adverse effects on the cardiovascular and pulmonary systems. Recent evidence indicates that the novel benzodiazepine remimazolam has minimal inhibitory effects on the circulation and respiration. However, the efficacy and safety of remimazolam versus etomidate in the induction of anesthesia are unclear.

**Objective:**

To further understand the potential of remimazolam in anesthesia induction, it is necessary to design a meta-analysis to compare its effects versus the classic safe anesthetic etomidate. The aim of this study is to determine which drug has more stable hemodynamics and a lower incidence of PIHO. Our study will also yield data on sedation efficiency, time to loss of consciousness, time to awakening, incidence of injection pain, and postoperative nausea and vomiting with the two drugs.

**Methods:**

We plan to search the Web of Science, Cochrane Library, Embase, PubMed, China National Knowledge Infrastructure, and Wanfang databases from the date of their creation until March 31, 2025. The language is limited to English and Chinese. The search terms are “randomized controlled trials,” “etomidate,” and “remimazolam.” The incidence of PIHO is the primary outcome measure. Secondary outcomes include depth of anesthesia after induction, sedation success rate, time to loss of consciousness, hemodynamic profiles, recovery time, incidence of injection pain, and postoperative nausea and vomiting. Reviews, meta-analyses, case studies, abstracts from conferences, and commentaries will not be included. The heterogeneity of the results will be evaluated by sensitivity and subgroup analyses. RevMan software and Stata software will be used for data analysis. We will evaluate the quality of included studies using version 2 of the Cochrane risk-of-bias tool. The confidence of the evidence will be assessed through the Grading of Recommendations, Assessments, Developments, and Evaluations system.

**Results:**

The protocol was registered in the international PROSPERO (Prospective Register of Systematic Reviews) registry in November 2023. As of June 2024, we have performed a preliminary article search and retrieval for further review. The review and analyses are expected to be completed in March 2025. We expect to submit manuscripts for peer review by the end of June 2025.

**Conclusions:**

By synthesizing the available evidence and comparing remimazolam and etomidate, we hope to provide valuable insights into the selection of anesthesia-inducing drugs to reduce the incidence of PIHO and improve patient prognosis.

**Trial Registration:**

PROSPERO CRD42023463120; https://tinyurl.com/333jb8bm

**International Registered Report Identifier (IRRID):**

PRR1-10.2196/55948

## Introduction

As medical care advances worldwide, the number of patients undergoing surgery under general anesthesia has increased [1]. Postinduction hypotension (PIHO) is a hemodynamic abnormality commonly observed during induction of general anesthesia [2], which is defined as hypotension occurring within 20 minutes after anesthesia induction or before surgical incision. The incidence of PIHO was reported to reach 45%-55% [3-5]. Common PIHO risk factors include methods and dosages of anesthesia, poor baseline condition of the patient, hypovolemia, and poor cardiac function [6]. Because circulatory reserves weaken with age, older patients are especially vulnerable to the development of PIHO [7]. Acute renal damage [8], neurocognitive dysfunction [9], and perioperative cardiovascular events [10] are all linked to PIHO. Moreover, PIHO has been associated with increased mortality (8.8%), intensive care unit admission (7.9%), and requirement of postoperative mechanical ventilation (20.7%) [8,11]. Therefore, it is important to find safer and more effective anesthesia-inducing drugs to avoid PIHO.

Most intravenous anesthetics have a direct inhibitory effect on circulatory function [12]. Previous studies have found that etomidate has minimal adverse effects on the cardiovascular and pulmonary systems [13-15]. Etomidate is considered to be a somewhat safer drug for the induction of anesthesia in patients undergoing heart surgery because of the reduced risk of hypotension [16,17]. However, the drawbacks of etomidate, including adrenocortical depression and myoclonus, have limited its clinical use [18].

Remimazolam is a novel drug in the benzodiazepine class [19], and its effects initiate and end more rapidly than achieved with the common benzodiazepine midazolam. Remimazolam can be continuously infused owing to its ultrashort-acting profile [20]. Additionally, its clearance is not dependent on liver or renal function because the drug is metabolized by tissue esterase [21,22]. Recent research has shown that remimazolam and propofol have comparable rates of sedative success but that remimazolam is associated with less respiratory and circulatory depression [23-25]. 

However, the evidence comparing remimazolam and etomidate for induction of general anesthesia is conflicting. Huang et al [26] found that the remimazolam group had a higher incidence of PIHO and a lower heart rate during induction than the etomidate group. However, some studies have found that low-dose remimazolam is hemodynamically more stable and has fewer adverse effects than etomidate in noncardiac and cardiac surgery [27,28].

Therefore, to further understand the potential of remimazolam, it is necessary to design a meta-analysis to compare the effects of remimazolam versus the classic, safe anesthetic etomidate in the induction of general anesthesia. These results can help to determine which drug has more stable hemodynamics and a lower incidence of PIHO. In addition, our study will yield data on sedation efficiency, time to loss of consciousness, time to awakening, incidence of injection pain, and incidence of postoperative nausea and vomiting with the two drugs.

## Methods

### Study Registration

This meta-analysis is registered in the international PROSPERO (Prospective Register of Systematic Reviews) registry (CRD42023463120). The protocol is reported according to the PRISMA-P (Preferred Reporting Items for Systematic Review and Meta-Analysis Protocols) guidelines [29] (Multimedia Appendix 1)

### Search Strategy

We plan to search the Web of Science, Cochrane Library, Embase, PubMed, China National Knowledge Infrastructure, and Wanfang databases from the date of their creation until March 31, 2025. The language is limited to English and Chinese. We will identify additional relevant studies by screening the references of related research and the International Clinical Trials Registry platform. Multimedia Appendix 2 displays the complete PubMed search strategy.

### Eligibility Criteria

Studies are considered eligible for inclusion in the systematic review and meta-analysis based on the Patients/Population, Intervention, Comparison, Outcomes, and Study design (PICOS) criteria as follows: patients aged 18 years or older undergoing surgery under general anesthesia, remimazolam as the intervention delivered at the induction of anesthesia compared to anesthesia induction with etomidate, primary outcome is the incidence of PIHO, and the effect of the intervention is assessed in randomized controlled trials.

### Study Selection

Two authors will independently determine the articles retrieved from the databases that are eligible for inclusion by screening the titles and abstracts of prospective eligible papers and then examining the full texts of the publications. Disagreements will be settled through dialogue with a third author. Studies will be selected according to the PRISMA selection process.

### Data Extraction

Two authors will independently extract the following information from the included studies: first author, year of publication, patient characteristics, sample size, dosage of remimazolam, type of surgery, incidence of PIHO, time to loss of consciousness, and recovery time. We will attempt to contact the corresponding author for raw data if the results are reported as the median and range. If there is no response, the median and range values will be converted to the mean and standard deviation according to the methods described by Hozo et al [30]. Any disagreements will be resolved through discussion with a third author.

### Risk of Bias Assessment

We will evaluate the quality of the included studies using version 2 of the Cochrane risk-of-bias tool [31]. The judged domains include the randomization process, deviations from intended interventions, missing outcome data, measurement of the outcome, and selection of the reported results. Each domain is judged as high, low, or some concern. 

### Statistical Analysis

The meta-analysis will be carried out using RevMan software and Stata software. For continuous data, we will use the mean difference and 95% CI, whereas for dichotomous data, we will calculate the risk ratio and 95% CI. The *I*^2^ statistic will be used to analyze study heterogeneity. When there is insignificant heterogeneity (*I*^2^<50%), a fixed-effects model will be used; otherwise, a random-effects model will be used. In addition, if high heterogeneity is encountered, sensitivity analysis or subgroup analysis will be performed to determine the underlying reasons. Methods for sensitivity analysis will include, but are not limited to, the single-study exclusion method and meta-regression. Subgroup analysis will be performed based on the final included studies, focusing on patient age, sex, type of surgery, intervention dosage, and other factors. When more than 10 studies are included, publication bias will be assessed using Stata software for the Egger test or Begg test [32]. For all analyses, two-tailed tests assessed at a significance level of *P*<.05 will be used. The Grading of Recommendations, Assessments, Developments, and Evaluations approach will be used to assess the confidence of the evidence [33].

## Results

The protocol was registered in the PROSPERO registry in November 2023. As of June 2024, a preliminary article retrieval step has been performed (see Multimedia Appendix 2). The review is expected to be completed by March 2025. We expect to submit manuscripts for peer review by the end of June 2025.

## Discussion

PIHO is more common in older patients owing to decreased vascular elasticity, autonomic nerve reflexes, and cardiac function reserve with age [34]. PIHO may lead to disastrous outcomes such as myocardial ischemia, cerebral infarction, kidney injury, neurocognitive impairment, and even death [35]. A recent large-sample (409 cases) multicenter randomized controlled trial found that induction of general anesthesia with remimazolam significantly reduced the incidence of PIHO and the use of vasoactive drugs compared with propofol. In addition, the incidence of bradycardia was significantly lower in patients in the remimazolam group [36].

Although etomidate has a relatively reduced effect on the circulation compared with other anesthesia drugs, several recent studies have found a lower incidence of PIHO with remimazolam than with etomidate [27,28,37]. However, these studies had small sample sizes and were single-center studies. Therefore, a meta-analysis is necessary to pool the results of the current studies. Many anesthetic drugs used during induction of anesthesia can trigger PIHO, including opioids. Considering the impact of opioids on PIHO, we will ensure that the same opioids are used across studies for comparison or we will perform subgroup analyses on this factor to help reduce the potential impact of opioids on the primary outcome of PIHO in our analysis.

This study also has some limitations. First, only articles published in English and Chinese are included in the search. Second, due to the novelty of this topic, a limited number of studies are available for analysis. Despite this limitation, it is necessary to synthesize the current studies on the incidence of PIHO during the induction of general anesthesia with remimazolam and etomidate to inform evidence-based clinical decision-making and plan future studies.

By synthesizing the available evidence and comparing the effects of remimazolam and etomidate, we hope to provide valuable insights into the selection of appropriate anesthesia-inducing drugs to reduce the incidence of PIHO and improve patient prognosis.

## Appendix

Multimedia Appendix 1 PRISMA-P (Preferred Reporting Items for Systematic review and Meta-Analysis Protocols) checklist.

Multimedia Appendix 2 Complete PubMed search strategy.

## Abbreviations

PICOS: Patients/Population, Intervention, Comparison, Outcomes, and Study design
PIHO: postinduction hypotension
PRISMA-P: Preferred Reporting Items for Systematic Reviews and Meta-Analyses Protocols
PROSPERO: Prospective Register of Systematic Reviews
