# Supplementary material for: Efficacy and Safety of Remimazolam Versus Etomidate for Induction of General Anesthesia: Protocol for a Systematic Review and Meta-Analysis
Source: JMIR Res Protoc. 2024 Jun 12;13:e55948. doi: 10.2196/55948 (PMC11208827; doi:10.2196/55948)
Supplement: Multimedia Appendix 2 [file resprot_v13i1e55948_app2.docx]

| **Search number** | **Query** | **Sort By** | **Search Details** | **Results** |
| --- | --- | --- | --- | --- |
| 4 | #1 AND #2 AND #3 | Most Recent | ("etomidate"[Title/Abstract] OR "Ethomidate"[Title/Abstract] OR "Radenarkon"[Title/Abstract] OR "Hypnomidate"[Title/Abstract]) AND ("Remimazolam"[Title/Abstract] OR "cns 7056"[Title/Abstract]) AND ("clinical study"[Title/Abstract] OR "controlled clinical trial"[Title/Abstract] OR "Randomized"[Title/Abstract]) | 7 |
| 3 | ((Clinical study[Title/Abstract]) OR (Controlled clinical trial[Title/Abstract])) OR (Randomized[Title/Abstract]) | Most Recent | "clinical study"[Title/Abstract] OR "controlled clinical trial"[Title/Abstract] OR "Randomized"[Title/Abstract] | 766,540 |
| 2 | (Remimazolam[Title/Abstract]) OR (CNS 7056[Title/Abstract]) | Most Recent | "Remimazolam"[Title/Abstract] OR "cns 7056"[Title/Abstract] | 551 |
| 1 | (((etomidate[Title/Abstract]) OR (Ethomidate[Title/Abstract])) OR (Radenarkon[Title/Abstract])) OR (Hypnomidate[Title/Abstract]) | Most Recent | "etomidate"[Title/Abstract] OR "Ethomidate"[Title/Abstract] OR "Radenarkon"[Title/Abstract] OR "Hypnomidate"[Title/Abstract] | 2,857 |
